# How *Drosophila suzukii* Acquires and Interacts with Its Microbiome Across Ecological Contexts

**DOI:** 10.3390/biology15100777

**Published:** 2026-05-13

**Authors:** Hunter Ernstberger, Gabriel Palmieri, Jennifer S. Sun

**Affiliations:** Department of Biochemistry and Microbiology, Rutgers University, 76 Lipman Drive, New Brunswick, NJ 08901, USA

**Keywords:** *Drosophila suzukii*, SWD, *Drosophila melanogaster*, niche, chemical ecology, insect microbiome, chemosensation, behavior, microbial associations, environmental biology

## Abstract

Spotted wing drosophila (*Drosophila suzukii*; SWD) has become a globally invasive pest by ovipositing in ripening, intact fruit rather than decaying material, a niche distinct from most other drosophilids. An expanding body of work implicates microbes and microbially derived chemistry as key drivers of this ecology, shaping fly biology across life stages. However, much of this evidence is derived from microbiome surveys and observational comparisons, further constrained by uncontrolled diet history, laboratory rearing, and insufficient ecological context. We examine how the SWD microbiome differs in which taxa are present (composition), how flies pick up those taxa from fruit and maternal sources (acquisition), how long those taxa are retained across life stages (persistence), and how each of these varies with diet, geography, season, and host crops. We then address how microbial cues and fermentation state function as context-dependent drivers of adult attraction, avoidance, and oviposition, and how microbe-mediated interspecific interactions reshape substrate suitability and competition among drosophilids. Throughout, we critically evaluate experimental designs and identify gaps that impede causal inference. These include limited strain-level resolution, incomplete fungal characterization, and weak linkages between microbial community structure and host phenotypes. Key unresolved questions include how SWD maintains performance across diverse hosts, how microbes modulate sensory processing during seasonal shifts, and which microbial metabolites drive attraction, avoidance, and competition. Resolving these questions is a direct prerequisite for field-stable integrated pest management (IPM), including microbially informed behavioral lures, oviposition deterrents derived from pathogen- and competitor-associated volatiles, and competitor-mediated suppression strategies. The experimental priorities identified here translate directly into a roadmap for the next generation of mechanistically grounded, ecologically realistic SWD management tools.

## Simple Summary

Spotted wing drosophila (*Drosophila suzukii*, or SWD) is an invasive fruit pest that damages raspberries, blueberries, cherries, and other soft fruits by laying eggs inside ripening, intact fruit before harvest. Unlike most of its relatives, which prefer rotting material, this fly targets fresh fruit, making it exceptionally difficult to manage and costly to growers worldwide. Current monitoring traps and management tools often perform inconsistently in orchards and vineyards, and a growing body of research suggests one reason: the microorganisms living in and around SWD play a much larger role in its biology than previously appreciated. Those microbial effects shift with fruit type, season, geography, and the fly’s physiological state. The same microbe can attract SWD in one setting and repel it in another, which helps explain why a lure that works in the lab may fail in the field. This review synthesizes what is known about how SWD acquires its microbiome and how those microbes shape its development and behavior. It identifies the experimental gaps that currently limit the design of reliable, field-stable management strategies. Closing these gaps is what will allow growers to move from broadly effective tools to microbially informed traps, deterrents, and biocontrol approaches tailored to specific crops and seasons.

## 1. Introduction

*Drosophila suzukii*, or spotted wing drosophila (SWD), has become a globally important crop pest that uses its hard, serrated ovipositor to lay eggs directly inside ripening fruit. This agriculturally deleterious behavior has motivated extensive research on insect chemosensation, host choice, and population management [1,2]. This niche specialization is supported by sensory and behavioral divergence from other drosophilids. SWD shows altered responses to sugar concentration, substrate texture, and bitter taste cues, all of which shape its oviposition choices [3,4]. Critically, ripening fruit itself is a microbial habitat. SWD’s niche, therefore, sits in proximity to a continuous supply of microbes that varies with fruit type, microclimate, and biocontrol agent use [5,6,7]. Microorganisms shape the chemistry of the fruit microenvironment and produce signals that guide adult foraging and oviposition. They also serve as nutrient sources for larvae and mediate interspecific interactions that reshape substrate suitability [8,9,10,11].

Microbial chemistry shapes adult attraction and oviposition behaviors, but its effects are highly contextual. A given set of microbial sensory cues can function as oviposition deterrents or attractants depending on the biological context. Odorant profiles may differ greatly with respect to microbe life-stage and metabolism, as observed in yeasts. Volatiles in substrate fruit headspace may even modulate behavioral responses to nearby microbial odorants by suppressing them [9,12,13].

An emergent direction of inquiry that suffers from this context problem concerns interspecific competition. SWD avoidance of competitor-associated substrates depends on shed competitor microbes and weakens when those microbes are manipulated [10,14]. Live competitor larvae deter SWD oviposition, while dead larvae do not, indicating that the relevant cues are generated dynamically during larval activity [15,16]. This points to new behavioral control levers, provided the proximate metabolites and their delivery dynamics can be identified.

Context is the central unresolved variable in current SWD microbial research and needs to be continuously reexamined and integrated into experimental design. Throughout this review, we use “context” in a deliberately structured sense, grouping the relevant factors into four categories. Biotic context refers to the microbial community itself, including strain identity, community composition, and competitor presence. Chemical context refers to the chemical environment in which microbial cues are produced and detected, including fermentation stage, volatile background, and host fruit headspace. Physiological context refers to the state of the fly, including sex, mating status, seasonal morph, and diet history. Experimental context refers to the conditions under which a finding was generated, including lab vs. field setting, gnotobiotic vs. conventional rearing, axenic baselines, and antimicrobial exposure. Subsequent sections use this scheme to specify which categories of context are relevant to a given finding, and apparent contradictions across studies are interpreted with reference to which contextual category was unmeasured or uncontrolled.

Two recurring interpretive limitations characterize the literature. First, behavioral studies that use defined chemical stimuli are entirely valid on their own terms, but their ecological interpretation is constrained when the microbial community, fermentation stage, or odor background of the source substrate is not characterized. This is particularly important when results are extrapolated to field-relevant fly responses. Second, microbiome surveys frequently describe community structure without linking results to specific drosophilid phenotypes such as larval development rate, survival, fecundity, attraction, or oviposition outcomes [17,18].

The aim of this review is to shift the central question of SWD microbial research away from which microbes are associated with the fly. Instead, we ask how microbial context (e.g., community composition, metabolic state, fermentation stage, and odor background) structures the ecological information SWD uses across its life cycle. Framing the literature this way clarifies where apparent contradictions reflect unmeasured context rather than conflicting biology, and points toward mechanistically grounded integrated pest management (IPM).

The review includes four themes. Section 2 examines what shapes the SWD microbiome, including how core taxa are defined, how composition varies across populations and seasons, the understudied role of fungi, and how flies acquire and retain microbes. Section 3 addresses why fruit-only diets reveal SWD’s nutritional dependence on microbes and where larval choice diverges from larval fitness. Section 4 develops the central argument that microbial cues mean different things in different contexts, examining seasonal and physiological modulation, yeast volatiles, taxonomic resolution, and the SWD-specific avoidance of microbe-associated substrates. Section 5 turns to microbially mediated competition, including competitor-associated avoidance, larval substrate modification, sour rot, and sensory divergence.

**Figure 1:**
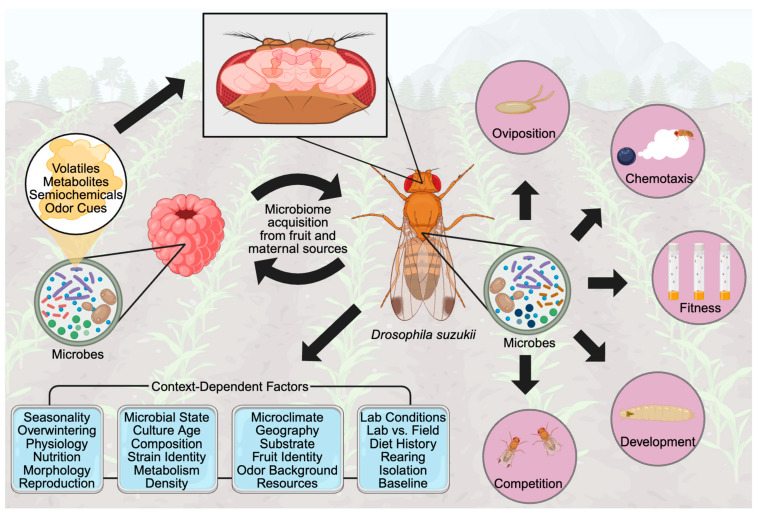
Microbial pathways shaping SWD biology and the contextual factors that modulate their interpretation. SWD acquires microbes from fruit substrates and maternal sources, and these communities influence oviposition, chemotaxis, larval fitness and development, and interspecific competition across the life cycle. Microbial effects are not fixed. They vary with fermentation stage, fruit type, odor background, season, host physiology, and strain identity, meaning the same microbe can carry different ecological meanings across contexts. Created with BioRender.com.

Figure 1 summarizes the conceptual framework of this review. SWD acquires microbes from fruit or maternal sources, and those microbial taxa can drive oviposition, chemotaxis, fitness, development, and competition. The figure emphasizes the manuscript’s central argument: microbial effects are context-dependent and must be interpreted accordingly. SWD is heavily influenced not only by the microbes it encounters, but also by which microbes are taken up, persist through development, and are sustained across life stages or ecological settings. Persistence may allow microbial taxa and their chemical influence to repeatedly shape host behavior and performance.

## 2. What Shapes the SWD Microbiome

### 2.1. Defining the SWD Core Microbiome

Defining “core” or “hub” taxa in SWD is difficult because the apparent composition of the SWD microbiome shifts substantially with the sampling method used (e.g., whole-body sequencing, gut-focused sampling, or fruit-surface metabarcoding), each of which captures a different subset of the microbial community [17,19]. Whole-body sequencing captures a mixture of gut, cuticle, reproductive tract, and transient environmental microbes [17,20,21,22]. Gut-focused sampling narrows this to the microbes most likely to influence nutrition and physiology [16,18,23,24,25]. Fruit-surface metabarcoding shifts the lens again, illuminating the microbial species on oviposition substrates and adult feeding sites that are quite often also detected in the fly microbiome [6,26,27]. Each approach answers a different question, and the apparent composition of the SWD microbiome changes accordingly.

Despite this methodological variability, recurring patterns do emerge. Survey-based studies of SWD-associated bacterial communities consistently report Pseudomonadota (formerly Proteobacteria), exemplified by *Tatumella* spp. and *Pantoea* spp., and a strong representation of acetic acid-producing bacteria (Acetobacteraceae, or AAB), such as *Acetobacter* spp. and *Gluconobacter* spp., across diverse diet contexts [19,22,24,25,28]. These are co-occurrence signals derived from 16S sequencing and metabarcoding, and detection by these methods does not by itself establish that the taxa are metabolically active, produce behaviorally relevant volatiles, or are present at physiologically significant absolute abundance. Within this caveat, AAB may contribute to semiochemical signaling for SWD based on olfactometer assays in which symbiotic AAB headspace elicits attraction [29]. Their role in natural fruit environments, however, remains unresolved and requires validation under more complex, field-relevant conditions.

The mechanistic chain from microbe to behavior is therefore partially resolved. AAB metabolize fruit sugars and ethanol to acetic acid and short-chain esters [29]. The headspace profiles dominated by these volatiles elicits attraction in laboratory assays [29], and AAB are detected in SWD-associated communities [19,22,24,25,28], but the link from laboratory attraction to in situ ecological function has not been established. Whether AAB-conditioned substrates support or reduce larval performance under realistic fruit conditions has not been systematically tested. This partial chain (microbe → metabolite → behavior, with fitness pending) typifies what the SWD field has and lacks for most candidate taxa.

Two caveats reinforce why family-level detection cannot be read directly as a functional signal. First, relative abundance differs from absolute load and does not necessarily correlate with metabolic activity. Microbiome studies need to quantify absolute load to capture true changes in microbial quantity [18,28]. Second, family-level patterns mask strain-level divergence in the microbiota. The same genus can include strains with different volatile profiles, varying growth on fruit substrates, and distinct effects on fly behavior and fitness, so family designations alone are often insufficient to determine function [20,29]. A recurring family in survey data does not, by itself, identify the strains doing the ecological work.

Moreover, fungal communities appear more substrate-linked and transient than bacterial communities, which have been shown to be more persistent members/taxa under certain lab-rearing conditions [30]. Recent field-scale and cross-region studies further show that any would-be “core” taxa depend on geography, crop context, sex, and season, and that “hub” taxa can shift with the sampling window [17,19,31]. Defining a single SWD microbiome therefore requires explicit spatial, temporal, and methodological boundaries, since core taxa identified under one set of conditions may not appear core under another.

One seasonal field study reported four core-associated genera that remained highly represented across seasons: *Gluconobacter*, *Pseudomonas*, *Commensalibacter*, and *Pantoea* [31]. Their persistence is notable because overall community structure shifted significantly within season in the same dataset. Some of these genera are common in plant-associated and environmental contexts. *Pseudomonas* and *Sphingomonas*, for example, appear in winter-associated or seasonally structured alimentary communities, consistent with a broader foraging niche outside of small fruit during colder months [32]. Seasonal shifts in olfactory preference between fruit and fermentation cues suggest that SWD interacts with distinct microbial habitats throughout the year [11,33,34,35]. The microbiome relevant to SWD biology is therefore likely seasonally structured and tied to seasonal resource use.

In summary, while recurring bacterial signatures (e.g., Pseudomonadota and acetic acid bacteria) emerge across SWD studies, these patterns are shaped as much by sampling methods, relative abundance metrics, and family-level resolution as by underlying biology. A defensible “core” microbiome thus requires explicit spatial, temporal, and methodological boundaries, alongside absolute load measurements and strain-level identification. Without these, recurring taxa risk being read as functional signals when they may instead reflect convergent sampling artifacts.

### 2.2. Drivers of Microbiome Variation Across Populations

Wild population surveys indicate that host crop and sampling location significantly shape microbiome composition and co-occurrence structure, while sex effects are stronger in network structure than in bulk composition [17]. Crop effects likely arise from two sources. First, fruits differ in chemistry and microbial colonization, including the relative abundance of yeasts attractive to SWD. Second, background fruit volatiles can amplify, mask, or interfere with the microbial odor cues flies use in foraging and oviposition decisions [6,12]. Both yeast and AAB isolates differ in headspace profiles and attractiveness, and yeast metabolic state can strongly shape odor output, suggesting that apparent species effects may sometimes reflect growth-stage differences [13,29,36]. Crop-driven differences in microbiome composition are thus inseparable from crop-driven differences in the chemical environment those microbes generate.

Sex and physiological status add another layer by regulating behavior through microbiome-dependent effects. Axenic females, for example, show reduced food-seeking behavior compared with conventionally reared females, consistent with microbiome-dependent modulation of foraging in a sex-specific manner [37]. Crop and sex effects, therefore, reveal both microbial community variation and shifts in behavioral and sensory priorities [11,17,37].

Geographic variation across regions is substantial in survey data [19,25], but few studies have separated geographic signal from the confounded effects of host fruit, microclimate, agricultural practice, insecticide exposure, and laboratory adaptation. Diet-driven shifts and persistent diet-history effects that indicate that apparent regional differences can equally reflect rearing exposure rather than geography per se have qualified this claim. The studies that come closest to disentangling these factors do so by either holding diet constant across collection sites or by tracking microbiome change after diet switches. Both approaches show substantial residual effects of prior exposure that persist across generations [38]. Until geographic comparisons routinely include shared-diet controls or cross-fostering across origins, “regional” differences in the SWD microbiome should be interpreted as joint signals of geography, host substrate, and rearing history rather than geography alone. A major need going forward is standardization, or at a minimum, detailed reporting that enables cross-study comparison, including diet composition, antimicrobial use, number of generations under each diet, and prior microbiome characterization.

These results indicate that microbiome variation across SWD populations is not driven by any single factor but by the interacting effects of host crop, geography, sex, physiological state, and rearing history. Crop and geographic effects are inseparable from the chemical environments those substrates generate, and apparent regional differences are confounded by diet history and antimicrobial exposure unless explicitly controlled. Standardized reporting of diet, generations under each diet, and antimicrobial use is therefore a prerequisite for meaningful cross-study comparison.

### 2.3. Fungi as the Missing Half of the SWD Microbiome

SWD interacts strongly with yeasts and filamentous fungi, yet fungal community research remains far less integrated into microbiome studies than bacterial work [5,6,39,40]. This imbalance matters because fruit-surface fungal communities vary strongly by fruit type, and the yeasts most attractive to SWD are not evenly distributed across hosts. One study found that attractive yeasts differ across fruit types and are most abundant on raspberries, including a high representation of *Hanseniaspora uvarum* relative to other fruits [6]. *H. uvarum* is one of the most extensively studied yeasts in SWD ecology [27,34,35,41,42,43,44,45,46], but its role is not universal. Reported effects on attraction, oviposition, and larval performance vary with fruit type, strain identity, fermentation stage, odor background, and the physiological state of the fly. The species should be understood as an important and frequently relevant taxon rather than a universal key symbiont across ecological contexts. These findings suggest that fruit identity shapes yeast community composition, which in turn influences the odor landscapes that drive SWD habitat use [12,13].

Fungal metabarcoding alone, however, cannot identify which strains produce key volatiles or whether the same yeast species is metabolically equivalent across fruits [5,13,36]. Comparative work on bacterial and fungal persistence further suggests that bacteria may be more consistently associated with SWD than fungi, which appear more transient and more indicative of the fruit substrate than of the fly itself [30]. This underexplored asymmetry has real potential to bias the field, because laboratory diets often include antimicrobials that disproportionately disrupt fungi, and fungal detection is sensitive to extraction and primer biases [30].

The consequences of this gap are most visible at the interface between microbiome surveys and behavioral work. Much of the behavioral literature depends on yeast identity and yeast metabolic stage, while many microbiome studies remain bacteria-centered. The organisms doing the behavioral work are often the ones least well characterized in community surveys. A critical next step is to pair bacterial and fungal profiling with volatile analysis from the same fruits and flies, so that community structure can be linked directly to chemical output and behavioral responses [12,13].

The asymmetry between bacterial and fungal characterization is not a minor methodological gap but a substantive interpretive problem. The organisms that do much of the behavioral work in SWD ecology (i.e., yeasts) are among the least well represented in community surveys. Closing this gap requires paired bacterial and fungal profiling alongside volatile analyses from the same fruits and flies, so that community structure can be directly linked to the chemical outputs and behavioral responses that motivate microbiome research in the first place.

### 2.4. How SWD Acquires and Retains Its Microbes

Experiments using fresh fruit matrices and wild isolates show that both fruit-associated microbes and maternal sources can contribute to larval microbiota [20]. The same work indicates that microbial identity matters for persistence, and that some yeasts can be detected in newly emerged adults after metamorphosis. This carry-over should not, however, be equated with stable vertical transmission or persistent symbiosis: persistence through metamorphosis appears to be partial and transient, varies with strain and substrate, and is readily overwritten by subsequent feeding exposures in adults [20,30,38]. This matters because SWD larvae develop inside fruit tissues, where microbial exposure is intricate, specific, and highly local. What ends up in a larva depends on where females place eggs, how small wounds and tissue breakdown develop in the fruit over time, and how microbes proliferate and spread within the fruit after oviposition [2,47]. Early colonizers can shape later community structure even when adult microbiomes shift again after new feeding exposures [20].

A recurring limitation across behavioral and fitness studies is that rearing history is rarely reported or controlled, even though microbiomes retain signatures of prior diets after switching to a common controlled diet [38]. This is critical for SWD because laboratory colonies are often maintained on standardized yeast-rich media that imprint microbiome baselines, and comparisons between wild-caught and colony-reared SWD therefore conflate microbiome, exposure, and genetic differences [38,39,40].

A more careful approach would be to characterize SWD microbiomes before assays, explicitly report diet composition and antimicrobial exposure across multiple generations, and use gnotobiotic or defined-microbe reconstitution with isolate panels drawn from relevant fruit contexts [8,20,29]. Without these controls, the field will continue to describe acquisition and persistence patterns that it cannot causally attribute to either the microbes themselves or to the prior diet, antimicrobial exposure, and rearing conditions that shaped the starting microbiome.

Acquisition and persistence in SWD are jointly shaped by maternal contributions, fruit-derived colonizers, and the rearing histories that precede any given experiment, yet these latter factors are rarely controlled or reported. Until microbiome characterization, diet composition, and multi-generational antimicrobial exposure are documented as standard practice, the field will continue to describe acquisition patterns it cannot causally attribute to either the microbes themselves or the histories that produced them. Gnotobiotic and defined-microbe reconstitution experiments using isolates from realistic fruit contexts are the most direct path forward.

## 3. Why Fruit Diets Make SWD Depend on Microbes

### 3.1. What Microbes Provide When Fruit Falls Short

Microbial effects on SWD performance under fruit-limited conditions can be productively separated into three mechanistic categories. Nutrient provisioning refers to microbial biomass acting as a direct nutrient source, supplying protein, lipids, and other macronutrients that fruit alone does not provide [8,48]. Nutrient processing and accessibility refers to microbial metabolism that releases or modifies nutrients within the fruit matrix, increasing what is available to the larva even where the microbes themselves are not consumed in bulk [8,49]. Metabolic signaling refers to microbially produced metabolites that modulate host physiology (e.g., altering immune response, growth signaling, or sugar-metabolism gene expression) independent of bulk nutritional content [21,49]. The same microbe can act through more than one of these pathways. The dominant mode often depends on fruit context and microbial abundance.

The strongest causal evidence that microbial effects on SWD depend on nutrient context comes from gnotobiotic reconstitution. In axenic vs. conventional comparisons, axenic SWD develop normally on nutrient-rich diets but show performance deficits in fruit diets such as strawberry or blueberry. This deficit can be rescued by reintroduction of defined microbial associations [8]. High-dose heat-killed microbes provide a partial rescue, consistent with nutritional provisioning. Microbial biomass acts as a nutrient source when resources are limiting. Live metabolism may still be required for full rescue depending on the fruit matrix and microbial community composition [8]. Larval success in intact fruit, therefore, depends not only on fruit macronutrients but also on microbial biomass availability and microbially mediated nutrient accessibility within the fruit matrix [8]. A complete model of SWD growth and performance must therefore consider all sources of nutrition in an infested fruit—sugars from fruit flesh, and protein and lipids from microbes [48,50].

This picture is further complicated by the dual role of some microbes as both commensals and sources of detriment. For example, *Acetobacter pomorum* is a *Drosophila*-associated gut microbe that can interfere with SWD immune responses and metabolism and produce harmful gluconic acid when flies are provided with a nutrient-rich diet [21]. This is an example of metabolic signaling producing a net cost rather than a benefit.

Targeted reintroduction experiments sharpen this picture. Direct manipulation provides the clearest causal link. When *Klebsiella oxytoca* was reintroduced into the midgut of axenic flies, SWD development accelerated and carbohydrate metabolism genes were enriched [49]. *K. oxytoca* appears to stimulate glycolysis and gluconeogenesis (a metabolic-signaling effect) and can also act as a key contributor to protein acquisition (a nutrient-provisioning effect), depending on bacterial quantity or biomass [49]. This illustrates that a single microbe can occupy multiple nutritional roles. The dominant mode shifts with abundance rather than being fixed by taxonomy.

These findings are often described as evidence of protein limitation [8,39], with axenic larvae on fruit-only diets showing developmental deficits that are partially rescued by yeast biomass. This pattern is conventionally interpreted as protein supplementation [8]. However, this bottleneck could equally reflect broader nitrogen and micronutrient constraints, with different microbes alleviating different constraints depending on fruit context [8]. Yeasts such as *Hanseniaspora* and *Saccharomyces* are rich in protein and lipids and likely contribute most to amino-acid and fatty-acid supplementation, particularly on fruits with low protein content [8,47]. Acetic acid bacteria, by contrast, are unlikely to function primarily as nutrient sources at typical fruit-substrate abundances, but may instead alter the chemical accessibility of fruit-derived nutrients through ethanol oxidation and acid production [29]. Lactic acid bacteria appear to occupy yet another role, modulating amino-acid availability through fermentation [49]. The fruit context determines which constraint is dominant: low-nitrogen fruits favor microbial contributions to nitrogen acquisition, while low-micronutrient or low-lipid fruits make biomass-rich yeasts the more important rescuers.

A critical next step is targeted nutrient supplementation paired with experiments that separate purified nutrient additions from intact microbial biomass, and that test whether live metabolism is required across specific fruit matrices [8,28,39]. Diet quality also interacts with density. Lower survival rates under overcrowded confinement are directly tied to poor diet substrates and the resulting competition in SWD, whereas the opposite is observed with better-quality feed [51]. The same study reported lower survival and slower development in low-protein diets compared with fruit or standard lab diets [51]. For SWD specifically, the literature still needs more attention to trade-offs: which microbes are beneficial in fruit contexts but detrimental in others, and whether those trade-offs interact with seasonal phenotype and shifting resource use.

### 3.2. When Larval Choice Diverges from Larval Fitness

Yeast-focused experiments suggest that SWD larvae may rely on yeasts for successful development, but larval preference does not always align with larval performance [40,50]. Larvae may prefer *H. uvarum* even when performance is more optimal on other yeasts [40], a mismatch consistent with volatile-driven preference that does not track nutritional value [13,36]. Choice and fitness are governed by different signals, and yeast identity alone does not predict which one will dominate.

Adult experiments show a parallel complexity. Different yeasts shape ingestion, fecundity, and survival, with species such as *H. uvarum* and *Saccharomycopsis vini* associated with preferential ingestion and higher fecundity in comparative work [39]. Physiological state layers onto this: mated females are more attracted to *H. uvarum* and blueberry odor sources in wind tunnel assays than their unmated counterparts, and when yeast was nearby, mated females oviposited fewer eggs on blueberries [52]. The same yeast cue can therefore promote attraction in one behavioral context and suppress oviposition in another, depending on reproductive state.

A significant gap underlying these results is that many experiments still do not control for the yeast’s metabolic stage. Yeast headspace and nutritional output change across growth phases. Studies comparing responses to differently aged *Saccharomyces cerevisiae* cultures show that drosophilids and tephritids differ in their sensitivity and preference to yeast volatile profiles in ways consistent with resource use [13]. Direct demonstration of metabolic-stage effects on behavior in SWD specifically is limited. The inference here is drawn primarily from broader drosophilid and tephritid work [13] and from the well-established physiology of yeast volatile production across growth phases. Until SWD-specific assays systematically vary yeast culture age while holding other contextual variables constant, metabolic stage will remain a plausible but unconfirmed determinant of behavior. Within this caveat, fermentation state and background fruit odors are still likely to determine whether yeast cues function as strong attractants, weak signals, or signals that are masked or behaviorally deprioritized [12,13]. Apparent species effects in this literature may therefore be partly, or even primarily, growth-stage effects.

## 4. Why Microbial Cues Mean Different Things in Different Contexts

### 4.1. Season and Physiology Reshape Cue Value

A consistent finding across SWD behavioral work is that microbial cues do not carry a fixed meaning [12,13]. Preference shifts across seasons and internal states can reverse what is attractive [11,32,33,53]. Protein-deprived females, virgin females carrying unfertilized eggs, and males show strong attraction to fermentation-associated cues, while fully fed reproductive summer-morph females tend to prefer fresh fruit cues [11]. Fermentation odors can therefore function as a food signal in one context and as a warning signal on an oviposition substrate in another [9,11]. The same chemistry, encountered by a different fly, means a different thing.

Axenic experiments add a mechanistic layer to this. The microbiome itself can shape foraging behavior in a sex-dependent way. Axenic females show reduced food-seeking behavior compared with conventionally reared females, supporting the idea that internal state and microbial association interact at the behavioral level [37]. Winter-form gut community patterns and winter ecology point in the same direction, suggesting a seasonal shift in microbial exposure and food resources that likely changes which microbial cues are even available in the field [32].

The clearest way forward is to pair sampling and assays across seasons, characterizing the same individuals for microbiome, odor environment, and behavioral response, rather than inferring microbial state from fruit stage alone [11,32,37]. Without this kind of paired design, the literature will continue to report behavioral differences that it cannot trace back to either the cue or the state that gave the cue its meaning.

### 4.2. Yeast Volatiles in Context

Yeasts are often implicated as strong drivers of SWD attraction, but the signal is rarely reducible to a single compound or species. Laboratory and field studies show that metabolic volatiles from *H. uvarum* and *Metschnikowia pulcherrima* can each attract SWD, and that combining them can increase field trapping, reinforcing the idea that blend composition and microbial pairing matter [26,27,34,36,41,42,43,44,45].

Blends are not automatically additive. Testing combinations of individually reported attractants shows that mixing known attractants can fail to produce attraction [54]. This may reflect masking effects, in which one volatile suppresses the perceptual or behavioral response to another. Or, this may reflect non-linear mixture rules, in which the response to a blend is not predictable from the sum or average of the responses to its components, but instead emerges from receptor-level interactions and competitive binding at olfactory neurons. Both phenomena are well-established in insect chemosensation more broadly and have direct implications for lure design in SWD [54]. Even classic fermentation lures used for monitoring and mass trapping, such as acetic acid and ethanol, rely on mixture logic rather than single-compound effects [55]. A small set of metabolites may dominate attraction under some conditions, while additional metabolites can blur the signal or shift behavioral output in others.

Odor background is a second major source of variation, and one of the clearest reasons attractions measured in the lab do not always translate to orchard environments [12]. Wind-tunnel experiments show that background fruit headspace can suppress attraction to symbiotic yeast cues [12]. The strength of this suppression depends on fruit type: raspberry odor produces strong inhibitory effects, while other fruit backgrounds do not under the same experimental conditions. Host plant volatile work shows the converse as well: intact fruit headspace can itself drive attraction in mated females and contains specific antennally active compounds that function as host cues [56]. Fruit and yeast cues are therefore not independent inputs but components of a single integrated odor landscape. Another source of background odor is competitors, since SWD have been shown to exhibit modulated responses to yeast volatiles in the presence of the *Drosophila melanogaster* sex pheromone Z4-11Al [57].

Layered onto this is the metabolic-stage problem: yeast volatile profiles change with culture age and metabolic state, and apparent species effects can sometimes reflect growth-stage effects [13], though, as noted there, the direct evidence in SWD remains limited and the inference rests largely on broader drosophilid and tephritid data. In the field, SWD is never responding to a single yeast headspace in isolation. It integrates yeast cues with host fruit odors and state-dependent priorities within a complex, shifting odor background [11,12,56].

Conflicting results across studies of *H. uvarum* and key acetic acid bacteria illustrate how unmeasured context can generate apparent contradictions. As a concrete example, two studies reported contrasting effects of *H. uvarum* on SWD attraction: Kleman et al. [58] documented strong specific attraction to *H. uvarum* in laboratory wind-tunnel assays using fresh yeast cultures and minimal odor background, whereas Huang and Gut [12] found that the same attraction was substantially suppressed when raspberry headspace was added as a background odor in otherwise comparable wind-tunnel conditions. The two outcomes are not contradictory once context is specified; the studies differ in chemical context (presence vs. absence of fruit headspace) and in experimental context (assay configuration), and reading them together identifies background fruit odor as a critical modulator of yeast attractiveness rather than a study-to-study inconsistency. Table 1 catalogs reported effects of SWD-associated microbes across study systems and highlights where context dependence (i.e., strain identity, culture age, host physiology, odor background) likely drives the variation.

### 4.3. Limits of Taxonomic Resolution

Symbiotic AAB can attract SWD through volatile metabolites, providing a bacterial semiochemical pathway that runs parallel to the better-studied yeast work and aligns with the frequent detection of Acetobacteraceae in SWD-associated microbiomes [18,19,22,24,25,28,29]. The problem is that this alignment can encourage broad taxonomic inferences that the underlying chemistry does not support. AAB differ in headspace composition and attractiveness depending on which strains are present and what their metabolic state is [29]. A microbiome survey that stops at family or genus can therefore confuse function with presence, because detecting AAB does not establish that the relevant metabolite-producing strains are present, active, and abundant in the specific odor context the fly is encountering [17,29].

Lactic acid bacteria (LAB) reinforce the same point from a different angle. Selection of LAB strains for improved trapping shows that strain choice and fermentation context can alter odor production and attractiveness, with some inoculated bait preparations eliciting electroantennographic responses and improved trapping outcomes compared with baseline bait [59]. These studies are typically framed as monitoring optimization, but they are mechanistically important: they demonstrate that microbial metabolism and growth context, not taxonomic identity alone, define behavioral outcomes. Strain and state are doing the work that family-level surveys assign to the family.

If microbiome differences are going to be interpreted as functional, then the field needs to know which metabolites and volatile organic compounds those microbes produce in realistic substrates and under realistic environmental conditions [13,17,29,59]. To move beyond this limitation, we propose four minimum standards for SWD microbiome research to support functional or behavioral inference. First, microbial identification should resolve to the strain, amplicon sequence variant, or genome level rather than stopping at the family or genus level. Strain-level divergence is consequential for volatile output and behavioral effect. Second, absolute microbial load should be quantified alongside relative abundance, because metabolic impact scales with absolute biomass. Third, volatile output should be characterized directly from the same material under conditions that approximate the substrate the fly encounters, rather than inferred from taxonomy. Fourth, behavioral or fitness validation should be performed on the same isolates, strains, or material from which composition and chemistry were measured, closing the loop from microbe to metabolite to host response. Studies meeting these four standards would substantially strengthen the field’s capacity to attribute SWD phenotypes to specific microbial drivers.

The tools to close this gap exist on the sensory side. Coeloconic sensilla mapping and functional characterization in SWD have produced species-specific sensory response landscapes that can be paired directly with microbial metabolite identification [60]. This offers a route from cue to receptor to behavior that family-level surveys cannot provide on their own.

### 4.4. Why SWD Avoids What Other Drosophilids Approach

One of the most consequential findings in SWD chemical ecology is that microbial growth can deter oviposition in this species [9]. This is striking because comparable microbial cues stimulate oviposition in other drosophilids, indicating a lineage-level reversal of how microbially associated substrates are evaluated. In oviposition-choice assays, SWD avoidance of microbe-associated substrates is strong under some conditions but is jointly modulated by overlapping substrate properties, such as hardness, skin thickness, shape, size, and texture [9,53,61,62,63,64,65,66,67]. These physical properties shape both the mechanical accessibility of the substrate to the ovipositor and the local microbial community that develops on or within it, so their effects on avoidance are coupled. This inversion aligns with SWD’s specialization in ripening fruit. Ripening fruit represents a window in which resources are available, and SWD experiences a different competitor and microbial context than at later, more fermented stages [14]. The ripening-fruit niche is consistent with a lineage-level shift in oviposition responses to microbially produced cues, in which microbial growth discourages egg laying rather than stimulating it [9].

A clear example of this pattern comes from pathogen-altered fruit odors. Blueberries infected with *Colletotrichum fioriniae* emit altered headspace profiles that correlate with reduced SWD oviposition in field surveys, and follow-up bioassays have experimentally identified specific esters as repellents [68]. SWD have also been shown to use olfaction to avoid the pathogenic fungus *Botrytis cinerea*, specifically when it is present on an oviposition substrate rather than on other parts of the substrate plant [69]. This supports the broader point that microbial state can generate aversive odor blends that SWD incorporates into oviposition decisions. What is unresolved is what these repellents mean to the fly: pathogen risk, reduced offspring value, increased competition, or simply altered fruit chemistry that no longer matches the ripening-fruit niche [9,68]. Distinguishing among these would require experiments that link microbial state, volatile output, and fitness outcomes within the same system, rather than treating repellency as a purely behavioral readout [68].

Table 1 catalogs the breadth of taxa studied and the context dependence of their reported effects, illustrating why apparent contradictions across studies often reflect unmeasured ecological context rather than conflicting biology.

**Table 1:** Microbial taxa reported to interact with SWD. Bacterial, fungal, yeast, candidate entomopathogen, and endosymbiotic taxa are listed with their classification, study system, response measured, reported effect on SWD, and key contextual factors that may modify interpretation. Apparent conflicts across studies often reflect ecological context rather than contradictory biology.

Microbe/Taxon	Group	Study System	Response Measured	Effect on SWD	Context Dependence	Study(s)
*Acetobacter* sp.	AAB	Lab	Oviposition	Neutral/aversive	Requires strain-level specificity	[9,70]
*Acetobacter cibinongensis*	AAB	Lab	Adult attraction	Neutral	Limited data	[29]
*Acetobacter persici*	AAB	Lab	Adult attraction	Neutral/aversive	Culture age	[29]
*Acetobacter pomorum*	AAB	Lab	Competitor-associated aversion	Neutral	Effect not significant	[10]
		Lab	Fitness	Delayed development and stunted body growth	Host species-specific	[21]
*Gluconobacter* sp.	AAB	Lab	Oviposition	Aversive	Requires strain-level specificity	[9]
*Gluconobacter cerinus*	AAB	Field	Adult attraction	Moderate attractant	Limited data	[26]
*Gluconobacter kanchanaburiensis*	AAB	Lab	Adult attraction	Attractive	Limited data	[29]
*Gluconobacter oxydans*	AAB	Lab Field	Adult attraction	Attractive	High selectivity in field with only moderate attraction	[26,29]
*Komagataeibacter hansenii*	AAB	Lab	Adult attraction	Neutral	Limited data	[29]
*Komagataeibacter saccharivorans*	AAB	Lab	Adult attraction	Attractive	Field validation required	[29]
*Klebsiella oxytoca*	Enterobacteriaceae	Lab	Fitness	Rescued development in a gnotobiotic line	Limited data	[49]
*Curtobacterium* sp.	Actinobacteria	Field	Adult attraction	Weak attractant	Limited data	[26]
*Colletotrichum fioriniae*	Entomopathogen	Lab	Adult attraction	Aversive	Field validation required	[68,71]
		Lab	Fitness	Decline in oviposition, fecundity, and increase in embryonic mortality	Spore-dependent	[71]
*Metarhizium robertsii*	Entomopathogen	Lab	Fitness	High mortality	No host species-specificity	[23]
*Xenorhabdus nematophila*	Entomopathogen	Lab	Fitness	Immune suppressive, larval mortality	Requires nematode symbiont	[72]
*Wolbachia pipientis*	Endosymbiont	Lab Field	Fitness	Fecundity, pathogen resistance	Limited data	[56,73,74]
*Lactobacillus* sp.	LAB	Field	Adult attraction	Moderately attractive	Requires strain-level specificity	[59]
*Lactobacillus brevis*	LAB	Lab	Competitor-associated aversion	Aversive	Interspecific host microbiota-mediated, effect rescued with altered association	[10]
*Leuconostoc pseudomesenteroides*	LAB	Lab	Fitness	Adult mortality	Limited data	[75]
*Oenococcus oeni*	LAB	Field	Adult attraction	Attractive	Requires strain-level specificity	[59,76]
*Pediococcus* sp.	LAB	Field	Adult attraction	Moderately attractive	Requires strain-level specificity	[59]
*Actinomucor elegans*	Fungi	Lab	Fitness	Overall beneficial	Limited data	[77]
*Botrytis cinerea*	Fungi	Lab	Adult attraction	Repellent	Presence on non-fruit, shows no effect, phytopathogen and candidate entomopathogen	[45,69]
*Geotrichum candidum*	Fungi	Lab	Fitness	Overall beneficial	Limited data	[77]
*Talaromyces minioluteus*	Fungi	Lab	Fitness	Overall negative	Limited data	[77]
*Candida* sp.	Yeast	Lab	Fecundity	Increase in oviposition	Requires strain-level specificity	[78]
*Candida californica*	Yeast	Lab	Adult attraction	Attractive	Physiological status matters, field validation required	[41]
*Candida zemplinina*	Yeast	Lab	Adult attraction	Attractive	Physiological status matters, field validation required	[34,36,41]
*Hanseniaspora opuntiae*	Yeast	Field	Adult attraction	Attractive	Limited data	[26]
*Hanseniaspora uvarum*	Yeast	Lab	Fitness	Substandard host development, nutritional relevance, stimulates oviposition	Preference-performance mismatch, requires field validation	[8,39,40,45,52,78]
		Lab Field	Adult attraction	Attractive	Background odor, host physiological status, medium	[12,35,36,41,44,45,58]
		Lab Field	Phagostimulation	Promotes phagostimulation	Effect is not consistent, mixture-dependent, sex-dependent	[39,42,43,52,79]
*Issatchenkia terricola*	Yeast	Lab Field	Adult attraction	Moderate attractant	Weaker in comparison to other yeasts	[35,41,44,45]
		Lab	Larval development	Performed adequately	Slower larval development time then other yeast counterparts	[39,40]
*Metschnikowia pulcherrima*	Yeast	Lab	Larval development	Decline in larval fitness	Preference-performance mismatch	[39,78,80]
		Lab Field	Adult attraction	Attractive	Mixture-dependent	[34,36,44]
*Pichia* sp.	Yeast	Field	Adult attraction	Weak attractant	Requires strain-level specificity	[26]
*Pichia kluyveri*	Yeast	Lab	Larval development	Performed adequately	Slower larval development time then other yeast counterparts	[40]
		Lab	Adult attraction	Moderate attractant	Limited data	[41]
*Saccharomyces cerevisiae*	Yeast	Lab	Fitness	Increase in oviposition, larval development, nutritional relevance, reduction in larval survival	Requires strain-level specificity, culture age, ethanol production	[13,40,45,78]
		Lab Field	Adult attraction	Moderate attractant	Weaker in comparison to other yeasts	[27,34,39,41,45,81]
*Saccharomycopsis vini*	Yeast	Lab	Adult attraction	Attractive	Field validation required	[44]
		Lab	Fitness	Increase in adult performance, neutral in larval survival	Limited data	[39,78]
*Starmarella bacillaris*	Yeast	Lab	Larval development	Decline in larval fitness	Limited data	[16]
*Wickerhamomyces pijperi*	Yeast	Lab	Adult attraction	Attractive	Mixture-dependent	[34,36]

## 5. Competition Is Microbially Mediated

### 5.1. Why SWD Avoids Competitor-Associated Substrates

Competition among drosophilids has historically been read as a behavioral phenomenon, but recent work shows that microbial mediation is doing much of the work [10]. Manipulative experiments establish microbial mediation directly. SWD avoids substrates associated with *D. melanogaster*, and this avoidance weakens when competitor-associated microbiota are experimentally manipulated, indicating that the cue is tied to microbial influence rather than to competitor presence as such [10]. Microbes, in other words, translate competitor presence into the chemical information SWD uses to judge whether a substrate is suitable for oviposition [10].

The mechanism is not yet reducible to a single inoculation. Direct application of microbes to media does not always robustly reproduce avoidance at low doses, suggesting that gut passage, microbial interactions, substrate chemistry, or microbial growth dynamics may be required to generate the relevant cue [10].

At least three non-exclusive explanations are consistent with this pattern. First, insufficient microbial abundance: direct inoculation may not reach the densities or growth rates achieved during in vivo larval activity, where competitor larvae continuously deposit and propagate microbes. Second, altered metabolism outside the host gut: microbes growing on media may produce different volatile and metabolite profiles than the same microbes processed through gut passage, where bile salts, host enzymes, and oxygen gradients shape downstream metabolism. Third, missing microbial interactions: the avoidance cue may depend on consortia rather than single isolates, with cross-feeding, succession, or quorum-dependent metabolite production required to generate the relevant chemistry. Distinguishing among these will require defined-consortium reconstitution, comparison of in-gut versus in-media metabolite profiles, and dose–response experiments at ecologically realistic abundances. Until the proximate metabolites are identified and a chemosensory mechanism is uncovered, similar avoidance patterns could in principle be reproduced by an assortment of candidate microbes, leaving the field able to describe interspecific competition between drosophilids without yet being able to attribute it to specific chemical signals [10].

Some SWD oviposition experiments point towards microbe-mediated competition effects even between conspecifics, usually in the form of host-marking, like scraping of the substrate or deposition of frass [82,83,84]. The nature of these host-marking pheromones (HMPs), however, is underexplored. A microbial origin for SWD HMPs has been proposed [83,84], but the supporting evidence is currently indirect and behavioral rather than direct and chemical. Behavioral assays show that frass and substrate scrapings from previously ovipositing females reduce subsequent oviposition by other SWD [83,84]. These effects parallel patterns seen in other drosophilid systems where microbially derived metabolites have been chemically identified [82]. The hypothesis that SWD HMPs are likewise microbial therefore rests on behavioral analogy to other systems and the plausibility of microbial chemistry as a deposition route. No manipulative or isolate-based study has yet linked specific compounds in SWD host-marking deposits to specific microorganisms, and identifying those compounds is a clear next step toward a complete model of SWD egg-laying behavior.

### 5.2. Deterrence Depends on Active Larval Modification

Deterrence in this competitive context is less about who was present, but about what they were doing [15]. Live *D. melanogaster* larvae and eggs have been shown to deter SWD oviposition, while dead larvae and cuticular hydrocarbon extracts do not, indicating that the relevant cue depends on active substrate modification during larval activity and oviposition [15,57,85,86]. That modification likely involves chemical changes to the substrate produced as larvae feed and move through it [15]. Competitor odors can shift SWD oviposition choices, and these effects interact with yeast cues, suggesting that SWD integrates fermentation signals and competitor-associated information rather than responding solely to yeast [57,59,85,86,87]. Oviposition in fruits shared with competitors often leads to poor outcomes for the eclosing SWD, so this is an essential behavioral cue [57,64,88,89,90]. SWD also engages in host marking as a social behavior which modulates intraspecific oviposition interactions [83,84]. Broader compilations of drosophilid interactions frame these findings as potentially useful for control, while emphasizing that the underlying mechanisms remain underexplored and that the chemical cues have not been directly identified [14].

The reciprocal direction matters as well. SWD-associated microbes can affect the development of other species: in gnotobiotic and isolate-based experiments, yeasts isolated from SWD contexts accelerate *D. melanogaster* larval development, while specific yeasts can slow it, showing that microbial identity carried by SWD can causally alter competitor performance [16]. SWD-associated microbes, therefore, contribute to substrate microbial states that shape how well competitors do on the same fruit, opening a feedback loop in which microbial identity influences competition outcomes in both directions [10,16]. Early SWD-associated microbial states could shift substrate quality in ways that later benefit other drosophilids, reframing the substrate as a dynamic microbial environment rather than a fixed competitive arena.

### 5.3. When SWD Damage Opens the Door for Others

Vineyard systems illustrate how SWD infestation and the fruit wounding it produces can facilitate disease outbreaks. Sour rot is a condition characterized by the production of acids within grape berries leading to the eruption of microbe-contaminated pulp which can be passed to other berries [91]. The disease is caused by a complex set of interactions between epiphytic yeasts, AAB, and drosophilids (despite grapes being a poor host for SWD [64,66,67]), which has wide-reaching implications for worldwide wine production [62,63,64,92]. This phenomenon links insect behavior directly to microbial spoilage and disease ecology [93].

One suggested model for disease etiology is that SWD, with its serrated ovipositor, permits the infestation of ripening fruits by other flies like *D. melanogaster* via mechanical damage. Being adapted to soft, rotting fruits, *D. melanogaster* needs SWD to provide an entry point via synergistic infestation like that also observed with *Zaprionus indianus* [93,94]. Other work, however, finds that single and combined effects vary across conditions [95]. Some experiments show limited facilitation of *D. melanogaster* infestation by SWD and stronger dependence on environmental and temporal factors, challenging the successive infestation hypothesis for sour rot outbreaks. These results are best read not as contradictions or outliers but as evidence that microbial ecology, the timing of injury, environmental conditions, and the stage of fruit and microbial growth together determine whether interspecific facilitation or competition dominates.

Regardless of the particular interactions between participating drosophilids, it appears that the underlying mechanisms behind sour rot require a tetrapartite set of interactions between macro- and microorganisms on grape berries [91,96]. Grape berries, especially those damaged by SWD oviposition or abiotic agents, allow for proliferation of epiphytic yeasts which feed on berry sugars and produce ethanol [63,91]. Drosophilids bring along AAB which convert the ethanol to acetic acid, which build up within the fruit and create sour rot symptoms [63,91,96,97]. Drosophilid larvae may also produce enzymes that aid in feeding on their host fruit which also contribute to acid production [91]. This links sour rot etiology back to the effects of active larval modification of substrates. Drosophilid activity also prevents fruit healing, which allows the infection to progress [91,96]. As drosophilids seem to be a necessary part of the onset of sour rot, studies have been performed to determine fruit qualities that make berries more susceptible to SWD infestation or vectoring, such as sugar content, skin thinness, and ripening time [62,63,64,92,98].

These traits map directly onto the context-dependent framework developed throughout this review. Sugar content shapes chemical context by determining which yeasts and AAB proliferate and how rapidly fermentation volatiles accumulate. Skin thinness shapes both physical access for ovipositing females and biotic context, since wounded fruit develops different microbial succession trajectories than intact fruit. Ripening time shapes the temporal dimension of microbial colonization, determining how mature the microbial community is when SWD encounters the berry. Vectoring potential is therefore not a fixed property of a fruit cultivar but a joint outcome of fruit chemistry, physical properties, and the microbial dynamics those properties enable. This framing is useful for cultivar-level susceptibility assessments aimed at IPM.

### 5.4. Where Microbial Cues Meet Sensory Divergence

SWD differs from many other drosophilids in how sugar, substrate shape, and mechanosensation contribute to egg-laying preference, and in evolutionary shifts in bitter-taste coding that reduce avoidance of ripening fruit substrates [3,4,61,99]. These advances are essential for interpreting field context, and they are beginning to be integrated with microbial-state evidence in both correlative and causal pathways. Microbial activity generates complex volatile blends, including acids and alcohols that are behaviorally relevant to SWD, and produces cues such as CO_2_ that can plausibly engage both olfactory and gustatory pathways [29,55,100]. These microbial products also shift quickly within a single fruit stage, depending on substrate condition, yeast growth phase, and bacterial community composition [9,13,29], meaning that the sensory system SWD has evolved is being asked to read a chemically unstable target.

Work on short-chain fatty acids (SCFAs) in *D. melanogaster* found that SCFAs had opposite effects on adults compared with larvae and relied on two larval-exclusive chemoreceptors, Or30a and Or94b [101]. SWD frequents unripe fruit with less SCFA production, and unlike *D. melanogaster*, shows reduced attraction to propionic acid and no triggered feeding behavior [101]. Brain imaging work has compared responses to ripe fruit, fermented fruit, leaves, and bacterial odors in *D. melanogaster* and SWD [102]. The two species differ structurally in the antennal lobes and in odor representation. However, no statistically significant behavioral comparisons were reported in this work. The link between the observed neural-representation differences and behavioral divergence remains an inference rather than a directly tested result. Connecting neural-representation differences to behavioral output will require paired neurophysiological and behavioral assays in the same individuals or controlled lines. Together, these findings suggest that SWD does not just encounter different microbial cues than its relatives. SWD processes them through a sensory system reweighted to match its niche.

CO_2_ can arise from both fruit respiration and microbial fermentation, and divergence in CO_2_ sensory responses within the SWD species group suggests that this cue is integrated differently than in *D. melanogaster* [100]. What CO_2_ means in SWD decision-making (i.e., ripening, fermentation, or a modulator that changes the value of other odors) remains unresolved. Answering it would require experiments that control microbial activity and fermentation stage while measuring receptor responses and behavior, rather than presenting CO_2_ as an odor in isolation [13,100].

## 6. Conclusions

Microbes are not incidental to SWD biology. They are active components of SWD ecology, shaping larval nutrition, adult foraging, oviposition decisions, seasonal behavior, and interspecific competition. Understanding SWD requires moving beyond broad descriptions of host fruit preference or microbiome composition alone, and instead treating ripening fruit as a dynamic microbial habitat in which chemistry, nutrient availability, and behavioral meaning are continuously reshaped.

Microbial effects are real but rarely universal. The same microbial cues that attract SWD adults to feed can also deter oviposition. Yeasts that support attraction may not maximize larval performance. Microbial associations that are beneficial in one nutrient context can be costly in another. These outcomes are further conditioned by fruit type, odor background, fermentation stage, physiological state, season, and rearing history.

The most important gap moving forward is the limited causal evidence linking microbial presence to host outcomes. Surveys have identified recurring bacterial and fungal taxa, but these patterns alone do not reveal which metabolites are produced, which sensory pathways are engaged, or which microbes alter fitness and behavior in field-relevant settings. Closing this gap will require strain-level resolution, absolute microbial load measurements, paired bacterial and fungal profiling, metabolomics and volatilomics, and direct behavioral and fitness assays in realistic fruit systems. Gnotobiotic and defined-reconstitution experiments, especially when combined with the receptor-level and neurophysiological tools already developed for SWD, will be essential. Apparent inconsistencies across the SWD microbiome literature reflect both unmeasured ecological context and methodological heterogeneity (i.e., assay design, rearing conditions, sampling approaches, reporting standards). Resolving the field will require improvements on both fronts.

Several findings already approach field readiness and warrant near-term applied attention. The strongest case is for yeast-based attract-and-kill formulations using strain-validated *H. uvarum* and *M. pulcherrima* blends, which combine documented field attraction with established mass-rearing protocols [42,43,79]. Pathogen-derived repellents, particularly the *C. fioriniae* ester suite, represent a second tractable lead, since the relevant volatiles are produced under realistic field conditions and can be deployed without live-microbe formulations [68]. Lactic acid bacterium-based liquid baits using strains such as *O. oeni* show field-validated improvements in trap captures and are closer to deployment than most other microbial leads [59,76]. Less mature but high-priority avenues include competitor-derived oviposition deterrents, where the proximate mechanism is not yet identified, and seasonally tuned monitoring lures that account for the morph-specific olfactory shifts documented across the year [11,33,34].

Within this broader agenda, four priorities stand out as both tractable and directly tied to field applications. First, strain-level profiling of microbial volatile production, paired with attraction assays against realistic fruit backgrounds, is needed to identify which metabolites drive SWD attraction. This is the foundation for microbially informed monitoring lures that remain effective across crops and seasons. Second, linked attraction, oviposition, and offspring-performance assays using identical microbial treatments and physiological states are required to resolve the preference–performance mismatches documented throughout this review, and to identify the cues that could be deployed as field-stable oviposition deterrents. Third, the proximate metabolites mediating competitor-associated oviposition avoidance must be identified through combined competitor assays, metabolomics, and microbial manipulation. This opens the door to competitor-mediated suppression strategies that do not require releasing live competitors. Fourth, seasonally paired sampling of microbiome composition, odor environment, and behavioral response is needed to determine how seasonal physiology reshapes cue interpretation, with direct implications for season-specific monitoring tools. Each of these priorities maps onto an unresolved question summarized in Table 2 and onto a specific IPM application, providing a research-to-application roadmap rather than a general call for further work.

Rather than asking which microbes are associated with SWD, the more productive question is how microbial processes structure the ecological information SWD uses to survive and proliferate. Answering that question is what will move the field from describing microbial presence to engineering microbially informed pest management.

## Figures and Tables

**Table 2:** Unresolved questions and experimental priorities in SWD microbial ecology. For each question, the table summarizes why it remains unresolved and proposes experimental approaches to move the field from correlational microbiome surveys toward causal, mechanistic, and field-relevant understanding.

Unresolved Question	Why It Remains Unresolved	Best Next Experiment
Which microbial taxa and their volatiles drive SWD attraction under field conditions?	Lab studies isolate microbial cues from fruit headspace, while community surveys identify taxa without linking them to volatile output or field-scale response.	Pair strain-level volatile profiling with attraction assays against defined fruit backgrounds; validate top candidates in orchard field trials.
Why are some microbial cues attractive for feeding but aversive for oviposition or poor for larval development?	Attraction, oviposition, and larval success are typically measured in separate systems, obscuring how physiology and substrate state shift cue interpretation.	Run linked attraction, oviposition, and offspring performance assays using identical microbial treatments, diet histories, and host physiological states; identify implicated pathways.
How do vertical vs. horizontal microbiome acquisition affect SWD chemosensory responses?	Transmission route is rarely linked to sensory outcomes, and microbiome history is seldom measured in chemosensory studies.	Compare gnotobiotic SWD with vertical-like vs. horizontal microbial exposure; assess behavior, microbiome persistence, and sensory responses at strain-level resolution.
Which metabolites mediate competitor-associated oviposition avoidance?	Competitor effects depend on substrate modification, but the proximate chemicals and microbial contributors driving deterrence remain poorly characterized.	Combine competitor assays with metabolomics, microbial manipulation, and sensory testing to identify deterrent compounds; test field-relevant application.
How do seasonal physiological changes alter sensory interpretation of microbial odors?	Seasonal physiology and odor response are rarely measured together; internal state is typically inferred from season rather than directly linked to cue interpretation.	Measure physiology, microbiome composition, and behavioral responses across seasonal morphs using a complex odor panel; add neurophysiological testing to elucidate sensory pathways.

## Data Availability

No new data were created or analyzed in this study. Data sharing is not applicable to this article.

## Abbreviations

The following abbreviations are used in this manuscript:

AAB	Acetic acid bacteria
HMPs	Host-marking pheromones
IPM	Integrated pest management
LAB	Lactic acid bacteria
SCFAs	Short-chain fatty acids
SWD	Spotted wing drosophila

## References

[1] Tait G., Mermer S., Stockton D., Lee J., Avosani S., Abrieux A., Anfora G., Beers E., Biondi A., Burrack H. (2021). *Drosophila suzukii* (Diptera: Drosophilidae): A Decade of Research Towards a Sustainable Integrated Pest Management Program. J. Econ. Entomol..

[2] Abraham J., Angeli S., Antwi J.B., Rodriguez-Saona C. (2022). Editorial: Research Advances on *Drosophila suzukii*. Front. Ecol. Evol..

[3] Wang W., Dweck H.K.M., Talross G.J.S., Zaidi A., Gendron J.M., Carlson J.R. (2022). Sugar Sensation and Mechanosensation in the Egg-Laying Preference Shift of *Drosophila suzukii*. eLife.

[4] Dweck H.K., Talross G.J., Wang W., Carlson J.R. (2021). Evolutionary Shifts in Taste Coding in the Fruit Pest *Drosophila suzukii*. eLife.

[5] Hamby K.A., Becher P. (2016). Current Knowledge of Interactions between *Drosophila suzukii* and Microbes, and Their Potential Utility for Pest Management. J. Pest Sci..

[6] Jones R., Fountain M.T., Andreani N.A., Günther C.S., Goddard M.R. (2022). The Relative Abundances of Yeasts Attractive to *Drosophila suzukii* Differ between Fruit Types and Are Greatest on Raspberries. Sci. Rep..

[7] Biasi A., Zhimo V.Y., Kumar A., Abdelfattah A., Salim S., Feygenberg O., Wisniewski M., Droby S. (2021). Changes in the Fungal Community Assembly of Apple Fruit Following Postharvest Application of the Yeast Biocontrol Agent *Metschnikowia fructicola*. Horticulturae.

[8] Bing X., Gerlach J., Loeb G., Buchon N. (2018). Nutrient-Dependent Impact of Microbes on *Drosophila suzukii* Development. mBio.

[9] Sato A., Tanaka K.M., Yew J.Y., Takahashi A. (2021). *Drosophila suzukii* Avoidance of Microbes in Oviposition Choice. R. Soc. Open Sci..

[10] Rombaut A., Gallet R., Qitout K., Samy M., Guilhot R., Ghirardini P., Lazzaro B.P., Becher P.G., Xuéreb A., Gibert P. (2023). Microbiota-Mediated Competition between *Drosophila* Species. Microbiome.

[11] Clymans R., Van Kerckvoorde V., Bangels E., Akkermans W., Alhmedi A., De Clercq P., Beliën T., Bylemans D. (2019). Olfactory Preference of *Drosophila suzukii* Shifts between Fruit and Fermentation Cues over the Season: Effects of Physiological Status. Insects.

[12] Huang J., Gut L.J. (2021). Impact of Background Fruit Odors on Attraction of *Drosophila suzukii* (Diptera: Drosophilidae) to Its Symbiotic Yeast. J. Insect Sci..

[13] Biasazin T.D., Herrera S.L., Kimbokota F., Dekker T. (2022). Diverging Olfactory Sensitivities to Yeast Volatiles Reflect Resource Partitioning of Tephritids and Drosophilids. Front. Ecol. Evol..

[14] Tungadi T.D., Powell G., Shaw B., Fountain M.T. (2023). Factors Influencing Oviposition Behaviour of the Invasive Pest, *Drosophila suzukii*, Derived from Interactions with Other *Drosophila* Species: Potential Applications for Control. Pest Manag. Sci..

[15] Tungadi T.D., Shaw B., Powell G., Hall D.R., Bray D.P., Harte S.J., Farman D.I., Wijnen H., Fountain M.T. (2022). Live *Drosophila melanogaster* Larvae Deter Oviposition by *Drosophila suzukii*. Insects.

[16] Solomon G.M., Dodangoda H., McCarthy-Walker T., Ntim-Gyakari R., Newell P.D. (2019). The Microbiota of *Drosophila suzukii* Influences the Larval Development of *Drosophila melanogaster*. PeerJ.

[17] Bhandari R., Wong A.C.-N., Lee J.C., Boyd A., Shelby K., Ringbauer J., Kang D.S. (2025). Microbiome Composition and Co-Occurrence Dynamics in Wild *Drosophila suzukii* Are Influenced by Host Crop, Fly Sex, and Sampling Location. Microbiol. Spectr..

[18] Lin Q., Zhai Y., Chen H., Qin D., Zheng L., Gao H. (2021). Analyses of the Gut Bacteriomes of Four Important *Drosophila* Pests. Can. Entomol..

[19] Medeiros M.J., Burger A.D., Price D.K., Yew J.Y. (2025). Microbiome Composition of *Drosophila suzukii* Varies across Geographical Regions. Front. Ecol. Evol..

[20] Guilhot R., Xuéreb A., Lagmairi A., Olazcuaga L., Fellous S. (2023). Microbiota Acquisition and Transmission in *Drosophila* Flies. iScience.

[21] Bing X.-L., Liang Z.-J., Tian J., Gong X., Huang S.-Q., Chen J., Hong X.-Y. (2024). The Influence of *Acetobacter pomorum* Bacteria on the Developmental Progression of *Drosophila suzukii* via Gluconic Acid Secretion. Mol. Ecol..

[22] Hiebert N., Carrau T., Bartling M., Vilcinskas A., Lee K.-Z. (2020). Identification of Entomopathogenic Bacteria Associated with the Invasive Pest *Drosophila Suzukii* in Infested Areas of Germany. J. Invertebr. Pathol..

[23] Hong S., Gao H., Chen H., Wang C. (2024). Engineered Fungus Containing a Caterpillar Gene Kills Insects Rapidly by Disrupting Their Ecto- and Endo-Microbiomes. Commun. Biol..

[24] Chandler J.A., James P.M., Jospin G., Lang J.M. (2014). The Bacterial Communities of *Drosophila suzukii* Collected from Undamaged Cherries. PeerJ.

[25] Martinez-Sañudo I., Simonato M., Squartini A., Mori N., Marri L., Mazzon L. (2018). Metagenomic Analysis Reveals Changes of the *Drosophila suzukii* Microbiota in the Newly Colonized Regions. Insect Sci..

[26] Bueno E., Martin K.R., Raguso R.A., Mcmullen J.G., Hesler S.P., Loeb G.M., Douglas A.E. (2020). Response of Wild Spotted Wing Drosophila (*Drosophila suzukii*) to Microbial Volatiles. J. Chem. Ecol..

[27] Lasa R., Navarro-de-la-Fuente L., Gschaedler-Mathis A.C., Kirchmayr M.R., Williams T. (2019). Yeast Species, Strains, and Growth Media Mediate Attraction of *Drosophila suzukii* (Diptera: Drosophilidae). Insects.

[28] Vacchini V., Gonella E., Crotti E., Prosdocimi E.M., Mazzetto F., Chouaia B., Callegari M., Mapelli F., Mandrioli M., Alma A. (2017). Bacterial Diversity Shift Determined by Different Diets in the Gut of the Spotted Wing Fly *Drosophila suzukii* Is Primarily Reflected on Acetic Acid Bacteria. Environ. Microbiol. Rep..

[29] Mazzetto F., Gonella E., Crotti E., Vacchini V., Syrpas M., Pontini M., Mangelinckx S., Daffonchio D., Alma A. (2016). Olfactory Attraction of *Drosophila suzukii* by Symbiotic Acetic Acid Bacteria. J. Pest Sci..

[30] Gurung K., Vink S.N., Salles J.F., Wertheim B. (2023). More Persistent Bacterial than Fungal Associations in the Microbiota of a Pest Insect. J. Pest Sci..

[31] Costa-Santos M., Sario S., Mendes R.J., Santos C. (2026). Seasonal Dynamics and Core Stability of the Bacterial Microbiome of a *Drosophila suzukii* Wild Population. Sci. Rep..

[32] Fountain M.T., Bennett J., Cobo-Medina M., Conde Ruiz R., Deakin G., Delgado A., Harrison R., Harrison N. (2018). Alimentary Microbes of Winter-Form *Drosophila suzukii*. Insect Mol. Biol..

[33] Kirkpatrick D.M., Leach H.L., Xu P., Dong K., Isaacs R., Gut L.J. (2018). Comparative Antennal and Behavioral Responses of Summer and Winter Morph *Drosophila suzukii* (Diptera: Drosophilidae) to Ecologically Relevant Volatiles. Environ. Entomol..

[34] Jones R., Goddard M.R., Eady P.E., Hall D.R., Bray D.P., Farman D.I., Fountain M.T. (2025). Differential Attraction of Summer and Winter Morphs of Spotted Wing Drosophila, *Drosophila suzukii*, to Yeasts. J. Chem. Ecol..

[35] Erdei A.L., Szelényi M.O., Deutsch F., Rikk P., Molnár B.P. (2022). Lure Design for *Drosophila suzukii* Based on Liquid Culture of Fruit Epiphytic Yeasts: Comparing the Attractivity of Fermentation Volatiles for Seasonal Morphs. J. Appl. Entomol..

[36] Jones R., Fountain M.T., Günther C.S., Eady P.E., Goddard M.R. (2021). Separate and Combined *Hanseniaspora uvarum* and *Metschnikowia pulcherrima* Metabolic Volatiles Are Attractive to *Drosophila suzukii* in the Laboratory and Field. Sci. Rep..

[37] Shu R., Hahn D.A., Jurkevitch E., Liburd O.E., Yuval B., Wong A.C.-N. (2021). Sex-Dependent Effects of the Microbiome on Foraging and Locomotion in *Drosophila suzukii*. Front. Microbiol..

[38] Jiménez-Padilla Y., Esan E.O., Floate K.D., Sinclair B.J. (2020). Persistence of Diet Effects on the Microbiota of *Drosophila suzukii* (Diptera: Drosophilidae). Can. Entomol..

[39] Spitaler U., Bianchi F., Eisenstecken D., Castellan I., Angeli S., Dordevic N., Robatscher P., Vogel R.F., Koschier E.H., Schmidt S. (2020). Yeast Species Affects Feeding and Fitness of *Drosophila suzukii* Adults. J. Pest Sci..

[40] Lewis M.T., Hamby K.A. (2019). Differential Impacts of Yeasts on Feeding Behavior and Development in Larval *Drosophila suzukii* (Diptera: Drosophilidae). Sci. Rep..

[41] Scheidler N.H., Liu C., Hamby K.A., Zalom F.G., Syed Z. (2015). Volatile Codes: Correlation of Olfactory Signals and Reception in *Drosophila*-Yeast Chemical Communication. Sci. Rep..

[42] Noble R., Dobrovin-Pennington A., Phillips A., Cannon M.F.L., Shaw B., Fountain M.T. (2019). Improved Insecticidal Control of Spotted Wing Drosophila (*Drosophila suzukii*) Using Yeast and Fermented Strawberry Juice Baits. Crop Prot..

[43] Bianchi F., Spitaler U., Castellan I., Cossu C.S., Brigadoi T., Duménil C., Angeli S., Robatscher P., Vogel R.F., Schmidt S. (2020). Persistence of a Yeast-Based (*Hanseniaspora uvarum*) Attract-and-Kill Formulation against *Drosophila suzukii* on Grape Leaves. Insects.

[44] Castellan I., Duménil C., Rehermann G., Eisenstecken D., Bianchi F., Robatscher P., Spitaler U., Favaro R., Schmidt S., Becher P.G. (2024). Chemical and Electrophysiological Characterisation of Headspace Volatiles from Yeasts Attractive to *Drosophila suzukii*. J. Chem. Ecol..

[45] Chakraborty A., Mori B., Rehermann G., Hernández Garcia A., Lemmen-Lechelt J., Hagman A., Khalil S., Håkansson S., Witzgall P., Becher P.G. (2022). Yeast and Fruit Fly Mutual Niche Construction and Antagonism against Mould. Funct. Ecol..

[46] Hamby K.A., Hernández A., Boundy-Mills K., Zalom F.G. (2012). Associations of Yeasts with Spotted-Wing Drosophila (*Drosophila suzukii*; Diptera: Drosophilidae) in Cherries and Raspberries. Appl. Environ. Microbiol..

[47] Woltz J.M., Lee J.C. (2017). Pupation Behavior and Larval and Pupal Biocontrol of *Drosophila suzukii* in the Field. Biol. Control.

[48] Bianchi F., Spitaler U., Robatscher P., Vogel R.F., Schmidt S., Eisenstecken D. (2020). Comparative Lipidomics of Different Yeast Species Associated to *Drosophila suzukii*. Metabolites.

[49] Gao H.-H., Zhao S., Wang R.-J., Qin D.-Y., Chen P., Zhang A.-S., Zhuang Q.-Y., Zhai Y.-F., Zhou X.-H. (2023). Gut Bacterium Promotes Host Fitness in Special Ecological Niche by Affecting Sugar Metabolism in *Drosophila suzukii*. Insect Sci..

[50] Silva-Soares N.F., Nogueira-Alves A., Beldade P., Mirth C.K. (2017). Adaptation to New Nutritional Environments: Larval Performance, Foraging Decisions, and Adult Oviposition Choices in *Drosophila suzukii*. BMC Ecol..

[51] Hardin J.A., Kraus D.A., Burrack H.J. (2015). Diet Quality Mitigates Intraspecific Larval Competition in *Drosophila suzukii*. Entomol. Exp. Appl..

[52] Mori B.A., Whitener A.B., Leinweber Y., Revadi S., Beers E.H., Witzgall P., Becher P.G. (2017). Enhanced Yeast Feeding Following Mating Facilitates Control of the Invasive Fruit Pest *Drosophila suzukii*. J. Appl. Ecol..

[53] Stockton D.G., Cha D.H., Loeb G.M. (2021). Does Habituation Affect the Efficacy of Semiochemical Oviposition Repellents Developed Against *Drosophila suzukii*? *Environ*. Entomol..

[54] Hasan K., Dweck H. (2025). Behavioral Responses of *Drosophila suzukii* to Blends of Its Attractants. microPubli. Biol..

[55] Landolt P.J., Adams T., Rogg H. (2012). Trapping Spotted Wing Drosophila, *Drosophila suzukii* (Matsumura) (Diptera: Drosophilidae), with Combinations of Vinegar and Wine, and Acetic Acid and Ethanol. J. Appl. Entomol..

[56] Revadi S., Vitagliano S., Rossi Stacconi M.V., Ramasamy S., Mansourian S., Carlin S., Vrhovsek U., Becher P.G., Mazzoni V., Rota-Stabelli O. (2015). Olfactory Responses of *Drosophila suzukii* Females to Host Plant Volatiles. Physiol. Entomol..

[57] Kwadha C.A., Rehermann G., Tasso D., Fellous S., Bengtsson M., Wallin E.A., Flöhr A., Witzgall P., Becher P.G. (2024). Sex Pheromone Mediates Resource Partitioning Between *Drosophila melanogaster* and *D. suzukii*. Evol. Appl..

[58] Kleman I., Rehermann G., Kwadha C.A., Witzgall P., Becher P.G. (2022). *Hanseniaspora uvarum* Attracts *Drosophila suzukii* (Diptera: Drosophilidae) With High Specificity. J. Econ. Entomol..

[59] Alawamleh A., Ðurović G., Maddalena G., Guzzon R., Ganassi S., Hashmi M.M., Wäckers F., Anfora G., Cristofaro A.D. (2021). Selection of Lactic Acid Bacteria Species and Strains for Efficient Trapping of *Drosophila suzukii*. Insects.

[60] Xue Q., Hasan K.S., Dweck O., Ebrahim S.A.M., Dweck H.K.M. (2025). Functional Characterization and Evolution of Olfactory Responses in Coeloconic Sensilla of the Global Fruit Pest *Drosophila suzukii*. BMC Biol..

[61] Akutsu J., Matsuo T. (2023). Oviposition Preference for Spherical Surfaces Is Shared among Multiple *Drosophila* Species except *D. melanogaster* (Diptera: Drosophilidae). Entomol. Sci..

[62] Tonina L., Giomi F., Sancassani M., Ajelli M., Mori N., Giongo L. (2020). Texture Features Explain the Susceptibility of Grapevine Cultivars to *Drosophila suzukii* (Diptera: Drosophilidae) Infestation in Ripening and Drying Grapes. Sci. Rep..

[63] Ioriatti C., Walton V., Dalton D., Anfora G., Grassi A., Maistri S., Mazzoni V. (2015). *Drosophila suzukii* (Diptera: Drosophilidae) and Its Potential Impact to Wine Grapes During Harvest in Two Cool Climate Wine Grape Production Regions. J. Econ. Entomol..

[64] Ibn Amor A., Kukorellyné Szénási Á., Németh C., Deutsch F., Kiss B. (2025). Assessing Wine Grape Cultivar Susceptibility to Spotted Wing Drosophila and *melanogaster*-Type *Drosophila* in Hungarian Vineyards: Effects of Berry Integrity and Insights into Larval Interactions. Insects.

[65] Rodriguez-Saona C., Cloonan K.R., Sanchez-Pedraza F., Zhou Y., Giusti M.M., Benrey B. (2019). Differential Susceptibility of Wild and Cultivated Blueberries to an Invasive Frugivorous Pest. J. Chem. Ecol..

[66] Cai P., Song Y., Yi C., Zhang Q., Xia H., Lin J., Zhang H., Yang J., Ji Q., Chen J. (2019). Potential Host Fruits for *Drosophila suzukii*: Olfactory and Oviposition Preferences and Suitability for Development. Entomol. Exp. Appl..

[67] Olazcuaga L., Rode N.O., Foucaud J., Facon B., Ravigné V., Ausset A., Leménager N., Loiseau A., Gautier M., Estoup A. (2019). Oviposition Preference and Larval Performance of *Drosophila suzukii* (Diptera: Drosophilidae), Spotted-Wing Drosophila: Effects of Fruit Identity and Composition. Environ. Entomol..

[68] Rering C.C., Quadrel A., Urbaneja-Bernat P., Beck J.J., Ben-Zvi Y., Khodadadi F., Aćimović S.G., Rodriguez-Saona C. (2023). Blueberries Infected with the Fungal Pathogen *Colletotrichum fioriniae* Release Odors That Repel *Drosophila suzukii*. Pest Manag. Sci..

[69] Cha D.H., Hesler S.P., Brind’Amour G., Wentworth K.S., Villani S., Cox K.D., Boucher M.T., Wallingford A., Park S.K., Nyrop J. (2020). Behavioral Evidence for Contextual Olfactory-Mediated Avoidance of the Ubiquitous Phytopathogen *Botrytis cinerea* by *Drosophila suzukii*. Insect Sci..

[70] Sato A., Yew J.Y., Takahashi A. (2023). Effect of Acetic Acid Bacteria Colonization on Oviposition and Feeding Site Choice in *Drosophila suzukii* and Its Related Species. bioRxiv.

[71] Urbaneja-Bernat P., Waller T., Rodriguez-Saona C. (2020). Repellent, Oviposition-Deterrent, and Insecticidal Activity of the Fungal Pathogen *Colletotrichum fioriniae* on *Drosophila suzukii* (Diptera: Drosophilidae) in Highbush Blueberries. Sci. Rep..

[72] Garriga A., Mastore M., Morton A., Pino F.G.D., Brivio M.F. (2020). Immune Response of *Drosophila suzukii* Larvae to Infection with the Nematobacterial Complex *Steinernema carpocapsae*-*Xenorhabdus nematophila*. Insects.

[73] Cattel J., Nikolouli K., Andrieux T., Martinez J., Jiggins F., Charlat S., Vavre F., Lejon D., Gibert P., Mouton L. (2018). Back and Forth *Wolbachia* Transfers Reveal Efficient Strains to Control Spotted Wing Drosophila Populations. J. Appl. Ecol..

[74] Hamm C.A., Begun D.J., Vo A., Smith C.C.R., Saelao P., Shaver A.O., Jaenike J., Turelli M. (2014). *Wolbachia* Do Not Live by Reproductive Manipulation Alone: Infection Polymorphism in *Drosophila suzukii* and *D. subpulchrella*. Mol. Ecol..

[75] Hiebert N., Kessel T., Skaljac M., Spohn M., Vilcinskas A., Lee K.-Z. (2020). The Gram-Positive Bacterium *Leuconostoc pseudomesenteroides* Shows Insecticidal Activity against Drosophilid and Aphid Pests. Insects.

[76] Ðurović G., Alawamleh A., Carlin S., Maddalena G., Guzzon R., Mazzoni V., Dalton D.T., Walton V.M., Suckling D.M., Butler R.C. (2021). Liquid Baits with *Oenococcus oeni* Increase Captures of *Drosophila suzukii*. Insects.

[77] Gao H.-H., Xu N., Chen H., Liu Q., Pu Q.-Y., Qin D.-Y., Zhai Y.-F., Yu Y. (2017). Impact of Selected Fungi from an Artificial Diet on the Growth and Development of *Drosophila suzukii* (Diptera: Drosophilidae). J. Asia-Pac. Entomol..

[78] Bellutti N., Gallmetzer A., Innerebner G., Schmidt S., Zelger R., Koschier E.H. (2018). Dietary Yeast Affects Preference and Performance in *Drosophila suzukii*. J. Pest Sci..

[79] Rehermann G., Spitaler U., Sahle K., Cossu C.S., Donne L.D., Bianchi F., Eisenstecken D., Angeli S., Schmidt S., Becher P.G. (2022). Behavioral Manipulation of *Drosophila suzukii* for Pest Control: High Attraction to Yeast Enhances Insecticide Efficacy When Applied on Leaves. Pest Manag. Sci..

[80] Anagnostou C., LeGrand E.A., Rohlfs M. (2010). Friendly Food for Fitter Flies?—Influence of Dietary Microbial Species on Food Choice and Parasitoid Resistance in *Drosophila*. Oikos.

[81] Knight A.L., Basoalto E., Yee W., Hilton R., Kurtzman C.P. (2016). Adding Yeasts with Sugar to Increase the Number of Effective Insecticide Classes to Manage *Drosophila suzukii* (Matsumura) (Diptera: Drosophilidae) in Cherry. Pest Manag. Sci..

[82] Keesey I.W., Koerte S., Retzke T., Haverkamp A., Hansson B.S., Knaden M. (2016). Adult Frass Provides a Pheromone Signature for *Drosophila* Feeding and Aggregation. J. Chem. Ecol..

[83] Tait G., Park K., Nieri R., Crava M.C., Mermer S., Clappa E., Boyer G., Dalton D.T., Carlin S., Brewer L. (2020). Reproductive Site Selection: Evidence of an Oviposition Cue in a Highly Adaptive Dipteran, *Drosophila suzukii* (Diptera: Drosophilidae). Environ. Entomol..

[84] Elsensohn J.E., Aly M.F.K., Schal C., Burrack H.J. (2021). Social Signals Mediate Oviposition Site Selection in *Drosophila suzukii*. Sci. Rep..

[85] Kienzle R., Rohlfs M. (2021). Mind the Wound!—Fruit Injury Ranks Higher than, and Interacts with, Heterospecific Cues for *Drosophila suzukii* Oviposition. Insects.

[86] Kidera H., Takahashi K.H. (2020). Chemical Cues from Competitors Change the Oviposition Preference of *Drosophila suzukii*. Entomol. Exp. Appl..

[87] Matsuo T. (2025). Asymmetric Oviposition Behaviour between *Drosophila suzukii* and *Drosophila subpulchrella* Suggests Competition for the Shared Niche in Their Native Range. Ecol. Entomol..

[88] Shaw B., Brain P., Wijnen H., Fountain M.T. (2018). Reducing *Drosophila suzukii* Emergence through Inter-Species Competition. Pest Manag. Sci..

[89] Shrader M.E., Burrack H.J., Pfeiffer D.G. (2020). Effects of Interspecific Larval Competition on Developmental Parameters in Nutrient Sources Between *Drosophila suzukii* (Diptera: Drosophilidae) and *Zaprionus indianus*. J. Econ. Entomol..

[90] Dancau T., Stemberger T.L.M., Clarke P., Gillespie D.R. (2017). Can Competition Be Superior to Parasitism for Biological Control? The Case of Spotted Wing Drosophila (*Drosophila suzukii*), *Drosophila melanogaster* and *Pachycrepoideus vindemmiae*. Biocontrol Sci. Technol..

[91] Hall M.E., Loeb G.M., Cadle-Davidson L., Evans K.J., Wilcox W.F. (2018). Grape Sour Rot: A Four-Way Interaction Involving the Host, Yeast, Acetic Acid Bacteria, and Insects. Phytopathology.

[92] Brilinger D., Arioli C.J., Werner S.S., Boff M.I.C. (2023). *Drosophila suzukii* Population Dynamics in Vineyards and Wine Cultivars Susceptibility. Rev. Bras. Frutic..

[93] Rombaut A., Guilhot R., Xuéreb A., Benoit L., Chapuis M.P., Gibert P., Fellous S. (2017). Invasive *Drosophila suzukii* Facilitates *Drosophila melanogaster* Infestation and Sour Rot Outbreaks in the Vineyards. R. Soc. Open Sci..

[94] Bernardi D., Andreazza F., Botton M., Baronio C.A., Nava D.E. (2017). Susceptibility and Interactions of *Drosophila suzukii* and *Zaprionus indianus* (Diptera: Drosophilidae) in Damaging Strawberry. Neotrop. Entomol..

[95] Entling W., Hoffmann C. (2020). Single and Combined Effects of *Drosophila suzukii* and *Drosophila melanogaster* on Sour Rot Development in Viticulture. J. Appl. Entomol..

[96] Barata A., Santos S.C., Malfeito-Ferreira M., Loureiro V. (2012). New Insights into the Ecological Interaction Between Grape Berry Microorganisms and *Drosophila* Flies During the Development of Sour Rot. Microb. Ecol..

[97] Ioriatti C., Guzzon R., Anfora G., Ghidoni F., Mazzoni V., Villegas T.R., Dalton D.T., Walton V.M. (2018). *Drosophila suzukii* (Diptera: Drosophilidae) Contributes to the Development of Sour Rot in Grape. J. Econ. Entomol..

[98] Baser N., Broutou O., Verrastro V., Porcelli F., Ioriatti C., Anfora G., Mazzoni V., Rossi Stacconi M.V. (2018). Susceptibility of Table Grape Varieties Grown in South-Eastern Italy to *Drosophila suzukii*. J. Appl. Entomol..

[99] Akutsu J., Matsuo T. (2022). *Drosophila suzukii* Preferentially Lays Eggs on Spherical Surfaces with a Smaller Radius. Sci. Rep..

[100] Gadau A., Mills S., Zhu Jiang X.Y., Li C., Svetec N., Xu Z., Li W., Nagel K.I., Zhao L. (2026). Molecular Evolution of CO_2_-Sensing ab1C Neurons Underlies Divergent Sensory Responses in the *Drosophila suzukii* Species Group. PLoS Genet..

[101] Depetris-Chauvin A., Galagovsky D., Chevalier C., Maniere G., Grosjean Y. (2017). Olfactory Detection of a Bacterial Short-Chain Fatty Acid Acts as an Orexigenic Signal in *Drosophila melanogaster* Larvae. Sci. Rep..

[102] Dumenil C., Yildirim G., Haase A. (2025). Differential Coding of Fruit, Leaf, and Microbial Odours in the Brains of *Drosophila suzukii* and *Drosophila melanogaster*. Insects.

